# Pathogenesis and antigenic characterization of a new East European subtype 3 porcine reproductive and respiratory syndrome virus isolate

**DOI:** 10.1186/1746-6148-6-30

**Published:** 2010-06-04

**Authors:** Uladzimir U Karniychuk, Marc Geldhof, Merijn Vanhee, Jan Van Doorsselaere, Tamara A Saveleva, Hans J Nauwynck

**Affiliations:** 1 Laboratory of Virology, Faculty of Veterinary Medicine, Ghent University, Salisburylaan 133, 9820 Merelbeke, Belgium; 2 Department of Health Care and Biotechnology, KATHO Catholic University College of South-West Flanders, Wilgenstraat 32, 8800 Roeselare, Belgium; 3 S.N. Vyshelesskij Institute of Experimental Veterinary Medicine, Briketa 28, 220003, Minsk, Belarus

## Abstract

**Background:**

Porcine reproductive and respiratory syndrome virus (PRRSV) is divided into a European and North American genotype. East European PRRSV isolates have been found to be of the European genotype, but form different subtypes. In the present study, PRRSV was isolated from a Belarusian farm with reproductive and respiratory failure and designated "Lena". Analyses revealed that Lena is a new East European subtype 3 PRRSV isolate. The main purpose of this investigation was to study the pathogenesis and antigenic characteristics of PRRSV (Lena).

**Results:**

Obvious clinical and virological differences were observed between the animals inoculated with a recent European subtype 1 PRRSV isolate (Belgium A) and animals inoculated with PRRSV (Lena). Three out of six pigs inoculated with PRRSV (Belgium A) had anorexia and low fever at 3, 4 and 5 days post-inoculation (dpi). High fever, anorexia and depression were prominent signs in most pigs inoculated with PRRSV (Lena) between 2 and 28 dpi. Four pigs out of ten died during the experiment. *Arcanobacterium pyogenes *was isolated from lungs of one animal that died, and *Streptococcus suis *was isolated from lungs of one animal that was euthanized. The difference in viral titres in sera from PRRSV (Belgium A) and PRRSV (Lena)-infected pigs was statistically significant (p < 0.05) at 7, 10, 14 and 21 dpi. The highest viral titres in sera ranged from 10^4.8 ^to 10^6.1 ^TCID_50_/ml for PRRSV (Lena) whereas they ranged from 10^3.1 ^to 10^4.8 ^TCID_50_/ml for PRRSV (Belgium A).

The replication of PRRSV (Lena) was further studied in depth. Viral titres ranged from 10^2.5 ^TCID_50_/100 mg to 10^5.6 ^TCID_50_/100 mg in nasal secretions between 3 and 14 dpi and from 10^2.8 ^TCID_50_/100 mg to 10^4.6 ^TCID_50_/100 mg in tonsillar scrapings between 3 and 21 dpi. High viral titres were detected in lungs (10^2.3^-10^7.7 ^TCID_50_/g tissue), tonsils (10^2.0^-10^6.2 ^TCID_50_/g tissue) and inguinal lymph nodes (10^2.2^-10^6.6 ^TCID_50_/g tissue) until 35, 28 and 35 dpi, respectively.

To examine the antigenic heterogeneity between the East European subtype 3 isolate Lena, the European subtype 1 strain Lelystad and the North American strain US5, sets of monospecific polyclonal antisera were tested in immunoperoxidase monolayer assays (IPMAs) with homologous and heterologous viral antigens. Heterologous antibody titres were significantly lower than homologous titres (p = 0.01-0.03) for antisera against PRRSV (Lena) at all sampling time points. For antisera against PRRSV (Lelystad) and PRRSV (US5), heterologous antibody titres were significantly lower than homologous titres at 14 and 21 dpi (p = 0.01-0.03) and at 10 and 14 dpi (p = 0.04), respectively.

**Conclusions:**

Lena is a highly pathogenic East European subtype 3 PRRSV, which differs from European subtype 1 Lelystad and North American US5 strains at both the genetic and antigenic level.

## Background

Porcine reproductive and respiratory syndrome virus (PRRSV) is a causative agent of reproductive failure in sows and respiratory disorders in pigs and had been firstly recognized in the USA in 1987 [1] and subsequently in Europe in the early 1990s [2]. The PRRSV genome contains nine open reading frames (ORFs) [3]. The ORF1a and ORF1ab code for proteins with apparent replicase and polymerase activities. Four minor structural proteins GP2, E, GP3 and GP4 are encoded by ORF2a, ORF2b, ORF3 and ORF4, respectively [3-5]. The major structural proteins are an envelope glycoprotein (GP5), an unglycosylated membrane protein (M) and a nucleocapsid (N) protein, encoded by ORFs 5, 6 and 7, respectively. PRRSV shows a high degree of genetic variation [6,7] and some antigenic heterogeneity [8-10]. Based on genetic and antigenic characteristics, PRRSV is divided into a European and North American genotype [11]. Recently, ORF5 and ORF7 sequences of East European PRRSV isolates were found to be of the European genotype, but group separately from all other European genotype sequences [12,13]. Stadejek and colleagues proposed a division of the European genotype into three subtypes: a pan European subtype 1 and East European subtypes 2 and 3 [13]. To our best knowledge, there is no information available concerning the pathogenesis of recent East European PRRSV isolates. Antigenic heterogeneity between European and North American PRRSV strains had been described earlier [8-10], however this issue remains to be determined towards new East European PRRSV subtypes.

The main purpose of the present study was to investigate the pathogenesis and antigenic characteristics of a recently isolated East European subtype 3 PRRSV isolate.

## Results

### Sequence information

The nucleotide sequences of ORF2a, 4, 5, 6 and 7 and deduced aa (amino acid) sequences were determined for the Belarusian PRRSV Lena (GenBank: EU909689, EU909690, EU909691, EU909692, EU909693) and North American PRRSV US5 (except ORF2a) (GenBank: EU926971, EU926972, EU926973, EU926974). All sequences were compared to the European reference strain Lelystad and the North American reference strain VR-2332 (Table 1). Phylogenetic analysis revealed that Lena is a new East European subtype 3 PRRSV isolate. Compared to previously described subtype 3 Belarusian isolates [12,13], PRRSV (Lena) showed between 87-96% and 90-98% identity for the GP5 and N proteins, respectively. The Lena GP2, GP4, GP5, M and N proteins were 249, 184, 201, 173 and 124 aa long. PRRSV (Lena) as well as other previously described subtype 3 Belarusian isolates had the lowest N protein size for the European genotype and the largest GP4 protein reported so far.

**Table 1:** Percentage of the GP2, GP4, GP5, M and N amino acid identity (pairwise comparison)

	**GP2**			**GP4**			**GP5**			**M**			**N**		
					
	**LV**	**US5**	**VR-2332**	**LV**	**US5**	**VR-2332**	**LV**	**US5**	**VR-2332**	**LV**	**US5**	**VR-2332**	**LV**	**US5**	**VR-2332**
					
Lena	92	NA	63	84	71	70	83	55	56	93	79	79	88	61	61
LV	100	NA	61	100	69	67	100	56	56	100	79	79	100	58	58
US5		100	NA		100	95		100	89		100	99		100	100

LV: Lelystad. NA: Not available.

### Clinical findings

**Figure 1:**
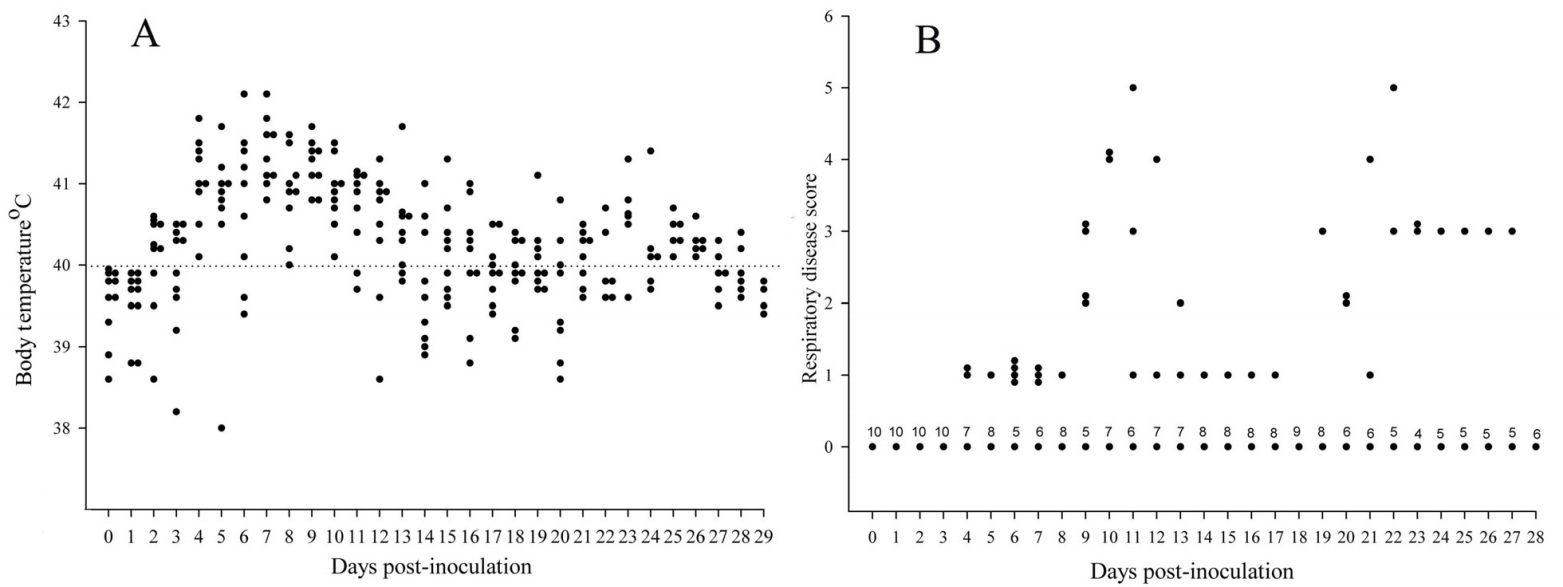
**Body temperature and respiratory disease scores in pigs inoculated with PRRSV (Lena)**. (A) Body temperature of pigs at different time points post-inoculation with PRRSV Lena. Temperature >40°C was considered as fever (dotted line). (B) The respiratory disease scores ranged from 0 to 6: **0 **= normal; **1 **= mild dyspnea and/or tachypnea when stressed; **2 **= mild dyspnea and/or tachypnea at rest; **3 **= moderate dyspnea and/or tachypnea when stressed; **4 **= moderate dyspnea and/or tachypnea at rest; **5 **= severe dyspnea and/or tachypnea when stressed; **6 **= severe dyspnea and/or tachypnea at rest. Stress was induced by holding the pig for 45 sec. The numbers above the dots represent the number of animals.

Obvious clinical differences were observed between the animals inoculated with PRRSV (Lena) and the animals inoculated with PRRSV (Belgium A). PRRSV (Lena)-inoculated pigs showed a rapid disease evolution. Anorexia and depression were seen in all animals from 5 to 18 dpi and periocular edema was observed from 3 to 9 dpi. Body temperature and respiratory disease scores are summarized in Figure 1. At 2 dpi, seven pigs out of ten had already fever which lasted until 28 dpi ranging from 40 to 42.1°C. Coughing was observed in some animals from 6 to 24 dpi. Four pigs died during the experiment; one at 4, one at 20 and two at 21 dpi. Out of six pigs inoculated with PRRSV (Belgium A), only three animals showed anorexia and fever (40°C) at 3, 4 and 5 dpi. Afterwards, temperature returned to normal, animals recovered and stayed healthy and alive until the end of the experiment. Respiratory disorders were not observed in these pigs.

### Gross lesions, bacteriology and differential diagnosis

The results of the postmortem and bacteriological examinations of pigs inoculated with PRRSV (Lena) are presented in Table 2. *Arcanobacterium pyogenes *was isolated from lungs of one animal that died, and *Streptococcus suis *was isolated from lungs of one animal that was euthanized. All pigs were negative for porcine circovirus 2, influenza virus, parvovirus and enteroviruses.

**Table 2:** Gross lesions in dead and euthanized pigs inoculated with PRRSV (Lena) and bacterial isolations

**Pig No**	Died^†^/euthanized* at ... dpi	**Gross lesions**	**Bacterial isolation**
	
1	4^†^	No visible gross lesions	-
			
2	20^†^	Fibrinous pleuropneumonia, fibrinous pericarditis	*Arcanobacterium pyogenes*
			
3	21^†^	Fibrinous pleuropneumonia, fibrinous pericarditis, peritonitis, enlarged bronchial lymph nodes	Negative
			
4	21^†^	Fibrinous pleuropneumonia, fibrinous pericarditis, peritonitis, enlarged bronchial lymph nodes	Negative
			
5	28*	Fibrinous pleuropneumonia, fibrinous pericarditis, peritonitis, enlarged bronchial lymph nodes	*Streptococcus suis*
			
6	28*	Fibrinous pleuropneumonia, fibrinous pericarditis, reactive spleen with follicular hypertrophy of the white pulp	Negative
			
7	35*	Fibrinous pleuropneumonia, fibrinous pericarditis	Negative
			
8	35*	Fibrinous pleuropneumonia, left diaphragmatic lobe mottled-tan with hemorrhage, right diaphragmatic lobe mottled-tan with hemorrhage and consolidation, fibrinous pericarditis, peritonitis	Negative
			
9	42*	Diaphragmatic lobes with sites of discoloration	-
			
10	42*	Diaphragmatic lobes with sites of discoloration, spleen reactive with follicular hypertrophy of the white pulp	-

-: A bacteriological investigation was not done.

### Viremia

**Figure 2:**
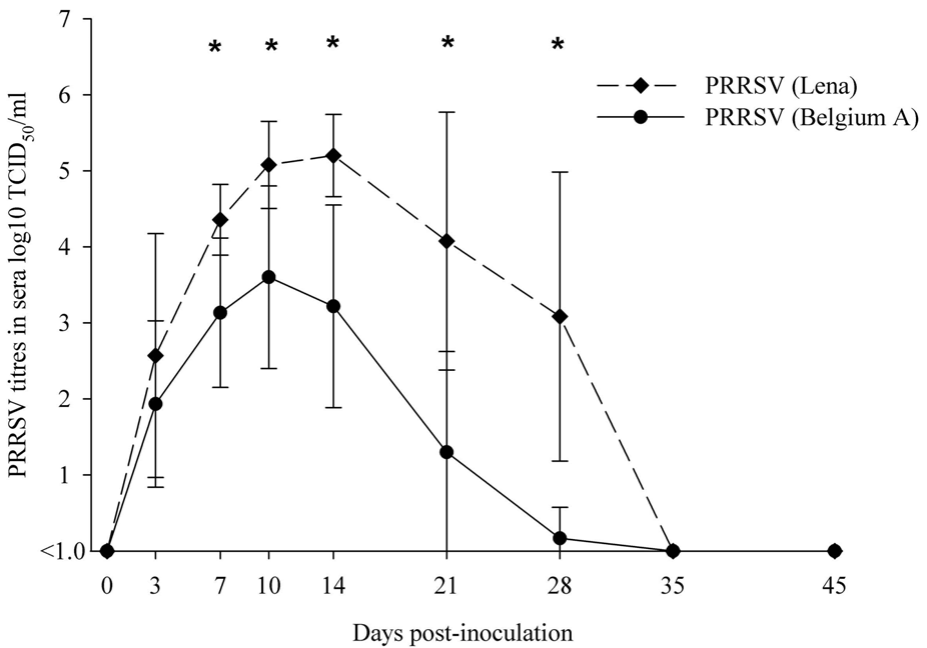
**Virus titres in sera of pigs at different days post-inoculation with PRRSV (Lena) and PRRSV (Belgium A)**. Symbols represent mean titres, whiskers above and below are standard deviations. Titres lower than 10^1.0^TCID_50_/ml were considered to be negative. *The difference is significant between virus titres.

Viral titres in sera of PRRSV (Lena)-inoculated animals were first detected at 3 dpi in nine pigs out of 10. At 7 dpi, all pigs had viremia which lasted until 28 dpi (Figure 2). The viral load in sera reached peak levels ranging from 10^4.0 ^to 10^6.1 ^TCID_50_/ml at 10 dpi and 10^4.3 ^to 10^6.0 ^TCID_50_/ml at 14 dpi. A high viremia was observed until 28 dpi. In pigs inoculated with PRRSV (Belgium A), viremia was detected from 3 dpi until 21 dpi. At 28 dpi only one pig out of six was still viremic with low viral titre (10^1.6 ^TCID_50_/ml). The viral titres in this group were significantly lower (p < 0.05) than in PRRSV (Lena)-inoculated animals at 7, 10, 14 and 21 dpi.

### PRRSV titres in different samples

PRRSV (Lena) was detected in nasal swabs from 3 dpi until 14 dpi and viral titres ranged from 10^2.5 ^TCID_50_/100 mg to 10^5.6 ^TCID_50_/100 mg. Tonsillar scrapings collected from all seven animals at 3 dpi had PRRSV titres ranging from 10^2.8 ^TCID_50_/100 mg to 10^4.6 ^TCID_50_/100 mg_. _At 14 dpi, five samples out of seven were positive with viral titres between 10^2.3 ^TCID_50_/100 mg and 10^3.6 ^TCID_50_/100 mg. At 21 dpi, three tonsillar scrapings out of five were PRRSV positive with viral titres ranging from 10^2.5 ^TCID_50_/100 mg to 10^3.1 ^TCID_50_/100 mg.

The results of virus titration of BAL (bronchoalveolar lavage) fluids and PAMs (porcine alveolar macrophages) are represented in Table 3. High viral titres were already detected in BALs and PAMs at 3 dpi. All BAL fluids and PAM samples were positive until 21 dpi. At 28 dpi, PAMs from only one pig were positive.

**Table 3:** Viral titres in BAL fluids and PAMs of pigs at different days post-inoculation with PRRSV (Lena)

**Pig No**	**PRRSV titres in BAL fluids (log10 TCID_50_/ml) at ... dpi**						**PRRSV titres in PAMs (log10 TCID_50_/10^6 ^cells) at ... dpi**					
		
	**3**	**14**	**21**	**28**	**35**	**42**	**3**	**14**	**21**	**28**	**35**	**42**
		
2	3.6	5.3					3.2	3.6				
3	3.5	5.8	2.6				4.2	2.7	4.0			
4	4.3	3.8	ND				4.2	2.8	ND			
5	5.1	6.8	7.8	<1.0*			4.2	3.0	4.3	<1.5*		
6	ND	ND	ND	<1.0*			ND	ND	ND	2.7*		
7	4.8	1.5	2.5		<1.0*		4.2	2.0	1.5		<1.5*	
8	4.8	5.6	3.0		<1.0*		4.2	2.7	1.8		<1.5*	
9	3.8	1.5	3.3			<1.0*	3.6	3.6	2.4			<1.5*
10	ND	ND	ND			<1.0*	ND	ND	ND			<1.5*

Pig 2 died at 20 dpi, pigs 3 and 4 at 21 dpi. *: Day of euthanasia. ND: not done.

High viral titres were detected in lungs (10^2.3^-10^7.7 ^TCID_50_/g tissue), tonsils (10^2.0^-10^6.2 ^TCID_50_/g tissue) and inguinal lymph nodes (10^2.2^-10^6.6 ^TCID_50_/g tissue) until 35, 28 and 35 dpi, respectively.

### Serology (IPMA results)

All animals were serologicaly negative prior to inoculation and at 0 and 3 dpi. Six out of nine PRRSV (Lena)-inoculated pigs seroconverted at 7 dpi and antibody titres ranged from 1.7 to 4.7 log4. From 10 dpi until the end of experiment all pigs were seropositive with antibody titres ranging from 4.7 to 7.7 log4. In animals inoculated with PRRSV (Belgium A), the course of anti-PRRSV antibody titres did not significantly differ from that in PRRSV (Lena)-inoculated pigs.

### Serological cross-reaction (IPMA results)

**Figure 3:**
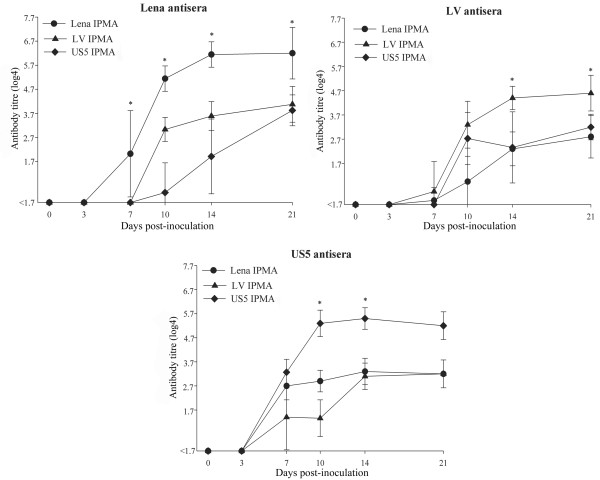
**The cross-reaction of antisera with homologous and heterologous IPMA antigens.** Symbols represent mean titres, whiskers above and below are standard deviations. Titres lower than 1.7 log4 were considered to be negative. *The difference is significant between homologous and heterologous antibody titres.

All samples collected at 0 and 3 dpi were seronegative regardless of the PRRSV used (Figure 3). Three PRRSV (Lelystad)-inoculated pigs out of ten seroconverted at 7 dpi and seven more at 10 dpi. All seven pigs inoculated with the PRRSV (US5) were seropositive at 7 dpi. The overall difference among the homologous antibody titres of the three groups was not significant (p = 0.1). All sera which were negative in the IPMA with homologous antigens were also negative in the IPMA with heterologous antigens. Heterologous antibody titres were significantly lower than homologous titres (p = 0.01-0.03) for antisera against PRRSV (Lena) at all sampling time points. For antisera against PRRSV (Lelystad) and PRRSV (US5), heterologous antibody titres were significantly lower than homologous titres at 14 and 21 dpi (p = 0.01-0.03) and at 10 and 14 dpi (p = 0.04), respectively.

## Discussion

In the present study, PRRSV was isolated from a Belarusian farm with reproductive and respiratory failure. It was designated Lena. The phylogenetic analysis revealed that PRRSV (Lena) is a new East European subtype 3 PRRSV isolate. The main purpose of this investigation was to study the pathogenesis and antigenic characteristics of PRRSV (Lena).

To study the pathogenesis, ten 6-week-old conventional pigs were inoculated with PRRSV (Lena). A fever, lasting for several weeks, and a range of respiratory and systemic clinical signs together with gross lesions such as severe fibrinous pleuropneumonia, pericarditis and peritonitis were observed, with four out of ten animals dying within three weeks post-inoculation. The clinical and pathological picture and high mortality are similar to what is seen in the field in Belarus (information from local veterinarians). A comparison of the findings obtained with PRRSV (Lena) with those obtained with other PRRSV strains revealed similarities with some highly pathogenic American strains [14]. Fever, prolonged viremia and co-infection with bacterial pathogens are prominent findings common to all of them. In this study, the viremia lasted for 4 weeks. As previously described for other PRRSV strains [15], antibodies produced early in infection were not effective in virus clearance. A good correlation was found between the extent of viremia and viral titres in nasal and tonsillar secretions and tissues.

The highest PRRSV (Lena) titres in sera in our investigation ranged from 10^4.8 ^to 10^6.1 ^TCID_50_/ml at different dpi. In the present study (European subtype 1 PRRSV isolate Belgium A) and in most previous studies, the highest PRRSV titres ranged from 10^3.6 ^to 10^4.8^TCID_50_/ml. To our knowledge, only few PRRSV strains had been reported to cause abnormally high viremia (10^5.2^-10^6.0 ^TCID_50_/ml) [16-19]. However, the animals used in those studies were younger (15-35 days of age) than in present experiment and in most studies only a small number of pigs had high PRRSV titres. Two younger pigs (four weeks of age) which were inoculated with PRRSV (Lena) had also higher viral titres in sera, 10^5.8 ^and 10^7.8 ^TCID_50_/ml (data not shown).

The severity of the observed clinical signs is most probably not due to a single direct effect of PRRSV (Lena). Secondary infections with other pathogens will have played an important role in the exacerbation of clinical signs. Indeed, in the present study *Arcanobacterium pyogenes *and *Streptococcus suis *were isolated from lungs of two pigs. It seems that PRRSV (Lena) infection paves the way for other pathogens. Co-infections of certain PRRSV strains with *Arcanobacterium pyogenes *as well as *Streptococcus suis *had been shown to increase the severity of the disease in pigs [14,20]. Damaging local respiratory macrophages potentially negatively affects the immune defense and allows bacteria to grow and to cause pathology and disease [21]. Pulmonary intravascular macrophages (PIMs) are thought to be very important in the clearance of circulating blood-borne bacteria in pigs [22]. The detrimental effects of PRRSV on PIMs have been demonstrated before and are considered as factors for the increased susceptibility to secondary infections [23-26]. The severity of disease caused by PRRSV (Lena) as a single agent should be verified in gnotobiotics.

The appearance of new highly virulent PRRSV strains and absence of effective diagnostic procedures and vaccines create a favorable environment for the emergence of devastating outbreaks. Pivotal examples are the recent atypical PRRSV outbreaks in China and Vietnam [27]. Sensitive and specific serological tests are important tools to control PRRSV. To characterize the antigenic heterogeneity between East European subtype 3 Lena, European subtype 1 Lelystad and North American US5 strains, sets of antisera were tested in IPMAs with homologous or heterologous viruses as antigens. Although the three sets of antisera reacted with all strains, the homologous antibody titres were always higher than the heterologous ones. The antigenic difference between the reference European strain Lelystad and Lena was similar to that between Lelystad and US5. Thus, East European subtype 3 Lena, European subtype 1 Lelystad and North American US5 PRRSV strains do share common antigenic determinants that induce production of antibodies reactive with the heterologous strains, but there are clear antigenic differences.

Since all present commercial kits for serological diagnosis are based on antigens belonging to European subtype 1 and North American PRRSV, it can be assumed that the observed heterogeneity might affect the accuracy of serological assays in geographical regions where East European Subtype 3 PRRSV strains circulate. Therefore, it is of major importance to validate the sensitivity of the current methods for serological PRRSV diagnosis in these regions.

## Conclusions

Lena is a highly pathogenic East European subtype 3 PRRSV, which differs from European subtype 1 Lelystad and North American US5 strains at both the genetic and antigenic level.

## Methods

### Virus isolation

Samples were collected from a nucleus herd of 5000 sows from the Eastern part of Belarus in January 2007. The herd experienced the following problems: abortion, birth of mummified, dead and weak piglets, high mortality rate before weaning, respiratory disorders and mortality (up to 70%) in growing pigs. The herd had been confirmed to be PRRSV positive. Also bacterial pathogens (*Streptococcus suis*, *Escherichia coli*) were regularly found in pigs with respiratory disorders. The lungs from weak-born piglets were collected for virus isolation. PAMs were obtained as previously described [28] from 4- to 6-week-old conventional Belgian Landrace pigs from a PRRSV-negative herd. PAMs were cultivated in RPMI 1640 modified medium (Gibco Invitrogen) supplemented with 10% fetal bovine serum (FBS), 2 mM l-glutamine (BDH Chemicals Ltd.), 1% non-essential amino acids (Gibco Invitrogen), 1 mM sodium pyruvate (Gibco Invitrogen), and a mixture of antibiotics in a humidified 5% CO_2 _atmosphere at 37°C. Subsequently, PAMs were inoculated with 20% suspensions of the lungs, CPE was observed and cells were fixed for 10 minutes with methanol (100%) at -20°C. The virus was identified by incubation with PRRSV-specific monoclonal antibodies P3/27 against the nucleocapsid protein [10] as previously described [29]. Then, the Belarusian isolate, designated Lena was grown in gnotobiotic macrophages and used for sequencing and the pathogenesis study. The virus stock was free from bacteria, pseudorabies virus, classical swine fever virus, parvovirus, enteroviruses and circoviruses.

### Sequencing and phylogenetic analysis

RNA was extracted using an RNeasy Protect Mini Kit (QIAGEN), according to the manufacturer's protocol. Total RNA was used as template in reverse transcription PCR with reverse transcriptase and random hexamer primers (Invitrogen) at 60°C annealing temperature and the cycling conditions were used as described by the manufacturer. PCR products were treated with Exonuclease I and Antarctic Phosphatase (New England Biolabs) and used directly for cycle sequencing with a Big Dye Terminator Cycle sequencing Kit V1.1 (Applied Biosystem) and PRRSV primers. Primers were designed based on the alignment of the genome sequences of strains BJ4, MLV, P129, VR2332 and Lelystad (GenBank: AF331831, EF484033, AF494042, EF442771, M96262, respectively). The primers are listed in Table 4. Cycle sequencing reaction products were purified by ethanol precipitation and separated on an ABI Genetic 310 (Applied Biosystem). In addition, the PCR products were electrophoresed on 5% agarose gels. PCR fragments of the correct size were cut out of the gel and purified using a QiaQuick gel extraction kit (QIAGEN). The purified fragments were sequenced as described above.

**Table 4:** Primers used for amplification and sequencing of PRRSV (Lena) and PRRSV (US5)

Target	Sequence	Location*	Direction	Target	Sequence	Location*	Direction
**Lena**				**US5**			
							
ORF2a	5'-gtsacaccktatgattacg-3'	11471-11489	Forward				
	5'-tcatrccctattytgcacca-3'	12642-12623	Reverse				
ORF4	5'-cggcccaittccatccigag-3'	12756-12775	Forward	ORF4	5'-cggcccaittccatccigag-3'	12756-12775	Forward
	5'-cattcagctcgcataicgtcaag-3'	13653-13631	Reverse		5'-cattcagctcgcataicgtcaag-3'	13653-13631	Reverse
ORF5	5'-tgcticatttcitgacacc-3'	13418-13436	Forward	ORF5	5'-tgcticatttcitgacacc-3'	13418-13436	Forward
	5'-accttaagigcitatatc-3'	14190-14173	Reverse		5'-accttaagigcitatatc-3'	14190-14173	Reverse
ORF6	5'-atgggaggcctagacgatttt-3'	14087-14107	Forward	ORF6	5'-taccaacttcattgtggac-3'	13922-13940	Forward
	5'-ccggccatacttgacgaggt-3'	14605-14586	Reverse		5'-acccagcaactggcacag-3'	14690-14673	Reverse
ORF7	5'-tggcccctgcccaicacg-3'	14412-14429	Forward	ORF7	5'-tggcccctgcccaicacg-3'	14412-14429	Forward
	5'-tcgccctaattgaataggtga-3'	15050-15030	Reverse		5'-tcgccctaattgaataggtga-3'	15050-15030	Reverse

*Locations are derived from Lelystad virus

The sequences were analyzed by BlastN and BlastP http://www.ncbi.nlm.nih.gov, and Sixframe, ClustalW and Align http://workbench.sdsc.edu.

### Adaptation of the PRRSV (Lena) isolate on MARC-145 cells

The use of PAMs in an IPMA for the detection of PRRSV antibodies in sera is difficult and expensive since cells have to be harvested from young pigs (preferably specific pathogen free animals). Certain monkey kidney cell lines (MA-104; MARC-145) can be a replacement for macrophages, but such cell lines do not support the growth of all isolates, particularly European ones. In order to get high virus replication in pigs and generate PRRSV (Lena) quasispecies which might favorably contribute to the adaptation on MARC-145 cells, two 4-week-old piglets from a PRRSV-negative farm were inoculated oronasally with 10^5 ^TCID_50_/pig of macrophage-grown virus. At 9 dpi, the pigs were euthanized and sera, lungs and tonsils were collected. Incubation of MARC-145 cells with suspensions of lungs and tonsils did not give positive results. However, incubation with sera resulted in virus replication which was visible by the presence of CPE (cytopathic effect) and was demonstrated by a PRRSV-specific staining. Subsequently, three passages on MARC-145 cells were necessary to further adapt PRRSV (Lena) and a virus stock with a titre of 10^4.6 ^TCID_50/_ml was obtained. The alignment of ORF(s) 2a, 4, 5 and 7 of the macrophage-grown and MARC-145 adapted Lena isolates revealed a 100% identity. This virus stock was used for the preparation of IPMA antigens.

### Animals and experimental design

At the age of six weeks, ten conventional pigs from a PRRSV negative herd were inoculated oronasally with 10^6 ^TCID_50_/pig PRRSV (Lena) in 2 ml of phosphate buffered saline (PBS, 1 ml in each nostril). Six pigs from the same origin were inoculated with 10^6 ^TCID_50_/pig of a recently isolated Belgian PRRSV, designated Belgium A, and served as a reference group for comparison of the clinical picture and virological findings. The Belgian PRRSV had been isolated from lungs and spleen of a stillborn piglet during a PRRSV outbreak in 2007. The herd experienced the following problems: birth of dead and weak piglets, high mortality rate among newborn piglets and respiratory disorders in growing pigs. The herd had been confirmed to be PRRSV positive. Sequencing demonstrated that PRRSV (Belgium A) belongs to the European subtype 1 PRRSV. The animal experiment was approved by the Ethical committee of the Faculty of Veterinary Medicine, Ghent University (EC2008/057).

Clinical signs (body temperature, respiratory disorders, and general signs such as appetite and behaviour) were monitored daily in both groups starting from six days before inoculation until the day of death or euthanasia. Respiratory disorders were scored from 0 to 6 (for interpretation, see legend Figure 1).

From the Lena-inoculated pigs, sera and nasal swabs were collected at 0, 3, 7, 10, 14, 21, 28, 35 and 42 dpi and stored at -70°C for virus titration. Also, sera were stored at -20°C for PRRSV-specific antibody detection. Seven out of ten Lena-inoculated pigs were anesthetized at 3, 14 and 21 dpi, for collection of tonsillar scrapings and *in vivo *pulmonary lavage as previously described [30]. The other three pigs were not treated and served as controls to exclude negative effects of *in vivo *pulmonary lavage. The lung lavages were centrifuged at 1500 × g for 10 min. The BAL fluids were collected, PAMs were resuspended in PBS and the total number of PAMs was calculated. Tonsillar scrapings, BAL fluids and PAMs were stored at -70°C for titration. At 28, 35 and 42 dpi, two pigs were euthanized and the following samples were collected: right lungs (apical, cardial and diaphragmatic lobes), tonsils and inguinal lymph nodes. In the case of death, the same organs were sampled. All samples were frozen and stored at -70°C for virus isolation and titration. The PAMs and BAL fluids were collected by pulmonary lavage from the left lung as previously described [31].

From the pigs inoculated with PRRSV (Belgium A), only sera were collected at the same time points as from PRRSV (Lena)-inoculated animals. All samples were stored at -70°C for virus titration and at -20°C for PRRSV antibody detection.

### Virus titration, serology and differential diagnosis

Sera, nasal swabs, tonsillar scrapings, BAL fluids, PAMs and tissue suspensions were titrated on PAMs from PRRSV negative pigs, as previously described [29]. For collection of nasal swabs and tonsillar scrapings, aluminum Rayon sterile plain swabs (COPAN, Italia) were used. Plain swabs were weighed before and after swabbing and viral titres were calculated per 100 mg of secretions. The monoclonal antibody P3/27 against the nucleocapsid protein was used for the detection of PRRSV-infected cells. PRRSV-specific antibodies were detected by an IPMA on Marc-145 cells as previously described [31]. PRRSV (Lena) and PRRSV (Belgium A) adapted on Marc-145 cells were used as IPMA antigens.

In the case of death or euthanasia, pigs were necropsied, lungs and heart were collected and bacteriological analyses were performed. All samples were inoculated on Columbia agar supplemented with 5% sheep blood (Oxoid, Hampshire, UK) with a *Staphylococcus pseudintermedius *streak for support of *Actinobacillus *and *Haemophilus spp*. growth, and on Columbia CAN agar with 5% sheep blood (Oxoid, Hampshire, UK). Plates were incubated overnight in a 5% CO_2_-inriched environment at 37°C and identification of isolated bacteria was performed as previously described [32]. PK-15, MDCK and CK cells were inoculated with 20% suspensions of lymph nodes and lungs for detection of circovirus 2, influenza virus and parvovirus/enteroviruses, respectively.

### Antigenic characterization

Sera from PRRSV (Lena)-inoculated pigs collected during the pathogenesis study were used. Two other panels of polyclonal antisera were obtained from six-week old pigs inoculated oronasally with 10^6 ^TCID_50_/pig of the PRRSV (Lelystad) [28] (10 pigs) or PRRSV (US5) (7 pigs). Blood samples were collected at 0, 3, 7, 10, 14 and 21 dpi. To examine the antigenic heterogeneity between East European subtype 3 Lena, European subtype 1 Lelystad and North American US5 strains, a serological cross-reaction study was undertaken and three panels of antisera were tested in IPMAs with Lena, Lelystad or US5 viruses as antigens.

### Statistical analysis

The difference between the overall courses of antibody titres obtained in three IPMAs with homologous antigens and between virus titres was analyzed by one way ANOVA. The differences between serological results in IPMAs with homologous and heterologous viral antigens at every sampling time point were assessed by non-parametric Wilcoxon matched pairs test. All statistical tests were performed with the STATISTICA 6.0 software. Differences were considered statistically significant at p ≤ 0.05.

## Authors' contributions

UUK carried out the isolation of PRRSV (Lena), the pathogenesis study, statistical analyses and antigenic characterization. He drafted the manuscript and participated in the study design, sequencing, phylogenetic analysis and in the adaptation of PRRSV (Lena) on MARC-145 cells. MG carried out the adaptation of PRRSV (Lena) on MARC-145 cells and participated in the pathogenesis study. MV participated in the antigenic characterization study. JVD carried out molecular genetic studies and phylogenetic analyses. TAS participated in the isolation of PRRSV (Lena). HJN conceived the study, coordinated the work and helped in writing the manuscript. All authors read and approved the final manuscript.
